# Effectiveness of Doxycycline in Combination With Other Antibiotics for Gram-Positive Periprosthetic Joint Infections: A Causal Inference Study

**DOI:** 10.1093/ofid/ofag098

**Published:** 2026-02-25

**Authors:** Jeanne Godon, Amadou-Khalilou Sow, Sylvia Das Neves, Coralie Humann, Alice Bordet, Ludovic Labattut, Thibault Sixt, Lucie Amoureux, Valentin Pineau, Christine Binquet, Sophie Mahy, Lionel Piroth, Mathieu Blot

**Affiliations:** Centre de Référence Interrégional Pour la Prise en Charge des Infections Ostéo-Articulaires Complexes (CRIOAc Dijon), Dijon, France; Département de Maladies Infectieuses, Hôpital Universitaire Dijon-Bourgogne, Dijon, France; CHU Dijon Bourgogne, Centre d’Investigation Clinique, Module Épidémiologie Clinique, INSERM, CIC1432, Université Bourgogne Europe, Dijon, France; Centre de Référence Interrégional Pour la Prise en Charge des Infections Ostéo-Articulaires Complexes (CRIOAc Dijon), Dijon, France; Département de Maladies Infectieuses, Hôpital Universitaire Dijon-Bourgogne, Dijon, France; Département de Rhumatologie, Hôpital Universitaire Dijon-Bourgogne, Dijon, France; Centre de Référence Interrégional Pour la Prise en Charge des Infections Ostéo-Articulaires Complexes (CRIOAc Dijon), Dijon, France; Service d’Orthopédie, Hôpital Universitaire Dijon-Bourgogne, Dijon, France; Centre de Référence Interrégional Pour la Prise en Charge des Infections Ostéo-Articulaires Complexes (CRIOAc Dijon), Dijon, France; Service d’Orthopédie, Hôpital Universitaire Dijon-Bourgogne, Dijon, France; Centre de Référence Interrégional Pour la Prise en Charge des Infections Ostéo-Articulaires Complexes (CRIOAc Dijon), Dijon, France; Département de Maladies Infectieuses, Hôpital Universitaire Dijon-Bourgogne, Dijon, France; Centre de Référence Interrégional Pour la Prise en Charge des Infections Ostéo-Articulaires Complexes (CRIOAc Dijon), Dijon, France; Laboratoire de Bactériologie, Hôpital Universitaire Dijon-Bourgogne, Dijon, France; Service des Maladies Infectieuses et Tropicales, Hôpital Universitaire de Nantes, Nantes, France; CHU Dijon Bourgogne, Centre d’Investigation Clinique, Module Épidémiologie Clinique, INSERM, CIC1432, Université Bourgogne Europe, Dijon, France; Centre de Référence Interrégional Pour la Prise en Charge des Infections Ostéo-Articulaires Complexes (CRIOAc Dijon), Dijon, France; Département de Maladies Infectieuses, Hôpital Universitaire Dijon-Bourgogne, Dijon, France; Centre de Référence Interrégional Pour la Prise en Charge des Infections Ostéo-Articulaires Complexes (CRIOAc Dijon), Dijon, France; Département de Maladies Infectieuses, Hôpital Universitaire Dijon-Bourgogne, Dijon, France; CHU Dijon Bourgogne, Centre d’Investigation Clinique, Module Épidémiologie Clinique, INSERM, CIC1432, Université Bourgogne Europe, Dijon, France; Centre de Référence Interrégional Pour la Prise en Charge des Infections Ostéo-Articulaires Complexes (CRIOAc Dijon), Dijon, France; Département de Maladies Infectieuses, Hôpital Universitaire Dijon-Bourgogne, Dijon, France; CHU Dijon Bourgogne, Centre d’Investigation Clinique, Module Épidémiologie Clinique, INSERM, CIC1432, Université Bourgogne Europe, Dijon, France; Center for Translational and Molecular Medicine (CTM), INSERM UMR1231, Lipness Team and LabEx LipSTIC, University of Burgundy, Dijon, France

**Keywords:** causal inference study, doxycycline, gram-positive bacteria, IPTW, periprosthetic joint infection, SBW, *Staphylococcus aureus*, TMLE, treatment failure

## Abstract

**Background:**

Doxycycline is occasionally used as step-down therapy in periprosthetic joint infections (PJI), but evidence supporting its efficacy is limited. This study aimed to estimate the effect of doxycycline on 12-month treatment failure in patients with gram-positive PJI.

**Methods:**

Adult patients with hip, knee, or shoulder PJI caused by *Staphylococcus*, *Corynebacterium*, or *Cutibacterium* who underwent surgery between April 2013 and April 2023 at Dijon University Hospital (France) were included. Demographic, clinical, biological, and therapeutic data were collected retrospectively. Treatment failure at 12 months was defined as clinical recurrence, new intraoperative microorganisms, surgical revision for infection, or death. The average treatment effect (ATE) of doxycycline was estimated using causal inference methods.

**Results:**

Three hundred and eighty-six patients with PJI (median age 72 years, interquartile range [IQR] = 65–80) were analyzed. Most infections involved the hip (62%) were caused by *Staphylococcus aureus* (64%) and/or polymicrobial (42%). Doxycycline was prescribed in 19% of patients (n = 72), for a median of 64 days (IQR = 42–84). At 12 months, treatment failure occurred in 35%, without significant difference between exposed and unexposed patients (33% vs 36%, *P* = .68). Overall, doxycycline was not significantly associated with treatment failure (ATE_(IPTW)_ = −0.09; 95% CI = −0.24 to 0.05; *P* = .19). Subgroup analyses suggested that doxycycline reduced treatment failure by 23%–26% in *S. aureus* infections (*P* < .001), 25%–28% in patients without fever (*P* < .001), and 34%–35% when both conditions were present (*P* < .001).

**Conclusions:**

Doxycycline in combination with other antibiotics was not associated with 12-month treatment failure in PJI caused by *Staphylococcus*, *Corynebacterium*, or *Cutibacterium,* with potential benefits in *S. aureus* infection warranting confirmation in prospective studies.

## Teaser

Doxycycline was not associated with overall treatment failure in gram-positive PJI, and subgroup analyses suggested reduced failure in *Staphylococcus aureus* infections, highlighting potential benefits that require confirmation in prospective studies.

Periprosthetic joint infections (PJIs) occur in ∼1%–4% of arthroplasty procedures, depending on the joint replaced [1]. Gram-positive bacteria are involved in more than three-quarters of PJI, including *Staphylococcus aureus*, coagulase-negative staphylococci, and *Cutibacterium* species [2, 3]. Management typically combines appropriate surgical intervention with prolonged antibiotic therapy, usually lasting 3 months [4]. The choice of oral follow-up antibiotic therapy is crucial, as it significantly impacts the prognosis. For staphylococcal PJI, rifampin is the first-line treatment, offering well-demonstrated anti-biofilm activity with improved clinical outcomes [5, 6]. Recommended oral companion drugs for rifampin include ciprofloxacin (grade A-I) or levofloxacin (grade A-II) [5]. Amoxicillin monotherapy is a suitable option for treating *Cutibacterium* species PJI [5, 7]. However, alternative agents are often required in cases of allergy, intolerance, or resistance to these first-line options. Among them, cotrimoxazole, linezolid or tedizolid, macrolides and related agents, and tetracyclines may be considered as second- or third-line therapies, although the level of evidence supporting their efficacy is considerably lower [5]. This is especially true for tetracyclines.

Consequently, doxycycline is seldom prescribed; in a large French multicenter cohort comprising 11,812 episodes, it was used in only 3.1% of cases, with the proportion rising to 14.5% when the PJI was due to resistant coagulase-negative staphylococci [2].

Doxycycline is an old, bacteriostatic tetracycline antibiotic characterized by excellent intestinal absorption and high oral bioavailability (95%–100%) [8, 9]. It achieves wide tissue distribution, notably in bone, where mean concentrations range from 1 to 5 μg/mL, above the MIC_90_ for *Staphylococcus* species [10]. Its low renal clearance makes it a preferred option for patients with renal comorbidities, reducing nephrotoxicity and drug accumulation. In addition, doxycycline exhibits activity against certain gram-negative bacteria, making it a putative option in polymicrobial infections, which account for approximately one-quarter of PJI cases [2]. Moreover, it is one of the most commonly used agents for suppressive antibiotic therapy in PJI in routine clinical practice, with a favorable safety profile even during prolonged treatment courses [11, 12].

In their systematic review of the literature, Cartau et al recently identified only 8 studies published between 1973 and 2024, with a maximum of 34 patients treated with tetracycline per study. In total, 62 patients received doxycycline as part of curative treatment for PJI, with reported success rates ranging from 82% to 100%, regardless of the surgical strategy [12]. None of the studies included a comparator arm to assess the efficacy of doxycycline against alternative treatments. The authors emphasized the low level of evidence supporting current recommendations for doxycycline use in PJI and highlighted the urgent need for well-designed studies [12].

The aim of this study was to evaluate the effect of doxycycline prescription compared with other strategies without doxycycline on the 12-month risk of treatment failure in patients treated for PJI involving at least 1 gram-positive bacterium. The secondary objective was to describe adverse events observed under doxycycline treatment.

## METHODS

### Design

We conducted a single-center retrospective cohort study at the CRIOAC (Referral Center for Complex Osteo-Articular Infections) Dijon University Hospital (France), including adult patients treated for PJI who underwent surgical management between April 1, 2013, and April 1, 2023.

### Patients

To be included, patients had to be older than 18 years, with PJI of the knee, hip, or shoulder as defined by the 2013 Infectious Diseases Society of America guidelines [5]. They must have undergone surgical intervention, either by arthrotomy with debridement or by prosthesis replacement. Microbiological documentation had to include at least one of the following gram-positive bacteria: *Staphylococcus* sp., *Corynebacterium* sp., or *Cutibacterium* sp., which represent the 3 main gram-positive pathogens for which doxycycline constitutes a relevant therapeutic option [5, 12–14]. For low-virulence organisms such as coagulase-negative staphylococci, *Corynebacterium* sp., and *Cutibacterium* sp., at least 2 positive intraoperative samples out of 5 collected were required to consider the culture clinically relevant, in accordance with standard PJI definitions [1].

Patients who received at least 5 days of doxycycline belonged to the exposed group, while patients who did not receive doxycycline were included in the unexposed group.

**Figure 1.**
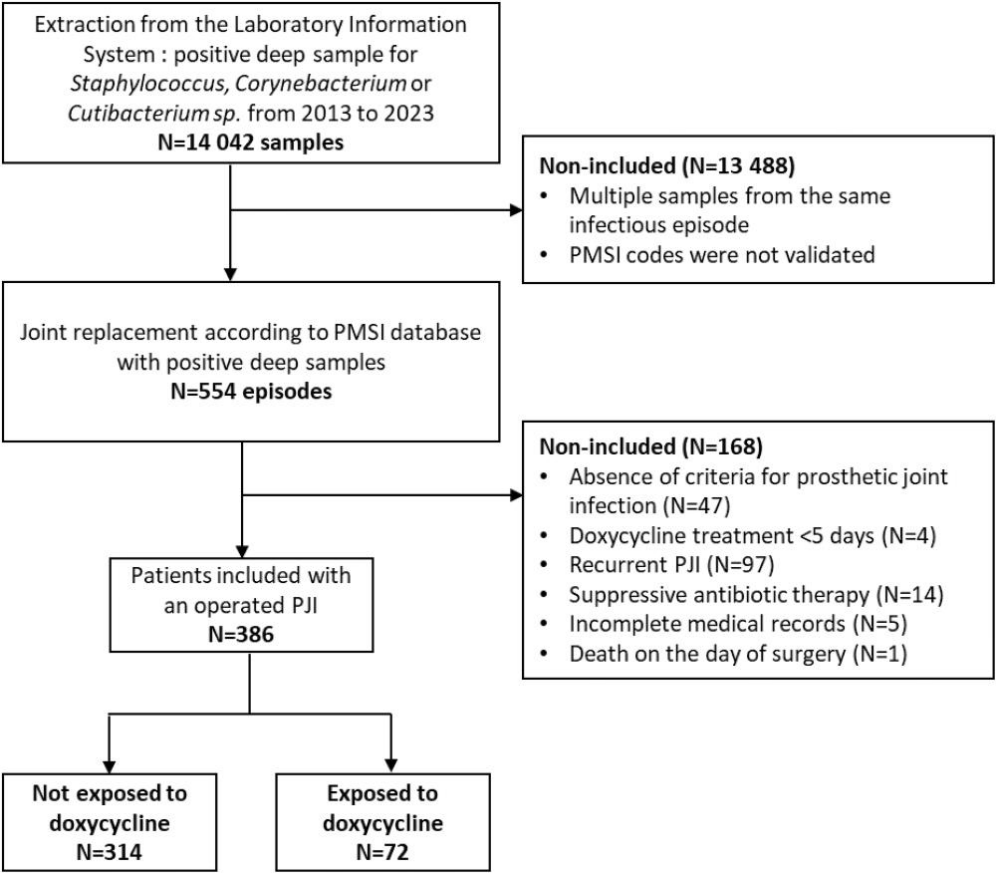
Flow chart. Abbreviations: PJI, prosthetic joint infection; PMSI, Programme de Médicalisation des Systèmes d’Information (French hospital discharge database).

Patients who received suppressive treatment decided at baseline, those who had previously undergone surgical management for the same infectious episode, those with incomplete medical records, or those who received doxycycline for <5 days were not included (Figure 1).

To identify eligible patients, the bacteriology laboratory extracted all deep osteo-articular samples that tested positive for *Staphylococcus*, *Cutibacterium*, or *Corynebacterium* species. This initial microbiological dataset was cross-referenced with the French hospital discharge database (PMSI) to retrieve relevant clinical and procedural information.

Cross-referencing was performed by extracting all hospital stays during the study period that met the following criteria: (1) the presence of ICD-10 code T84.5 (infection and inflammatory reaction due to internal joint prosthesis), (2) either as a primary or associated diagnosis, and (3) a corresponding surgical procedure involving the shoulder, hip, or knee joint, coded 13.03.02, 14.03.02, or 14.03.03, respectively, according to the French Common Classification of Medical Procedures.

### Outcomes

Treatment failure was defined by the occurrence of any of the following events: clinical recurrence of infection; isolation of new microorganisms during intraoperative samples (whether identical or different from the initial pathogens); surgical revision due to infection; and death.

Specifically, patients who were initially treated with a curative strategy but subsequently had a treatment failure and were continued on suppressive therapy during follow-up were considered as treatment failures rather than being excluded from the analysis.

Adverse effects reported during doxycycline treatment were collected.

### Data Collection and Definitions

For each patient included, demographic data, comorbidities (Charlson comorbidity index, chronic kidney disease, diabetes, and immunosuppression), clinical data as recorded in medical reports (fever at diagnosis, defined as a body temperature ≥38 °C in the first 48 hours following hospital admission and before surgery, local inflammation, purulent wound drainage, and presence of a sinus tract), biological data, and microbiological data (blood cultures, number of samples taken, and culture of deep samples) were collected. We also recorded the PJI type according to different classifications (early, delayed, late, and acute or chronic), as well as the surgical management (arthrotomy with debridement with or without exchange of mobile components, 1-stage prosthesis exchange, 2-stage prosthesis exchange, or other management) and antibiotic therapy (initial empirical and subsequent targeted treatment), with particular attention to doxycycline (doses, duration).

We defined infection chronicity based on the interval between the presumed date of inoculation and the date of diagnosis: acute (≤4 weeks) versus chronic (>4 weeks). The presumed inoculation date were considered to be the prosthesis implantation date for postoperative infections, and the symptoms onset or documented bacteremia dates for hematogenous infections. Additionally, the time from prosthesis implantation to infection onset was used to categorize infections as early (≤3 months), delayed (3–12 months), or late (>12 months) [1].

Surgical strategies were considered optimal, if they met the following criteria: (1) debridement, antibiotic therapy, and implant retention (DAIR) with exchange of mobile components for acute PJI; and (2) 1- or 2-stage prosthesis exchange for chronic PJI [15]. Any other surgical approach was classified as nonoptimal. To account for changes in clinical practices over time, we included the variable “management between 2018 and 2023” in comparison to the earlier period “2013–2017.”

### Ethics

The study protocol and data collection complied with French good practice regulations (Data Protection Act no. 78-17 of 6 January 1978) and European regulations (GDPR EU 2016/679) regarding data protection and patient information (Commitment of compliance MR004 no. 2210228 of 3 December 2018). Written patient consent was not required for this noninterventional study.

### Statistical Analyses

Categorical variables were presented as frequencies and percentages, and continuous variables were reported as medians with interquartile ranges (IQRs). Between-group comparisons were performed using Fisher's exact test or the χ^2^ test for categorical variables, and Student's *t*-test or the Wilcoxon rank-sum test for continuous variables, as appropriate.

The marginal effect of doxycycline exposure on 12-month treatment failure was estimated using a causal inference approach based on inverse probability of treatment weighting (IPTW) [16]. The propensity score was derived by logistic regression including confounders identified through a minimally sufficient directed acyclic graph (DAG) using Dagitty [17] (age, sex, diabetes, fistula, surgery, prosthetic site, and period (before/after 2018)), C-reactive protein, and polymicrobial infection [18]. If the initial propensity score model based on logistic regression failed to achieve adequate covariate balance (defined as standardized mean differences exceeding 0.1), an alternative weighting approach—stable balancing weights (SBW)—[19] was applied using the WeightIt package in R [20].

As a sensitivity analysis to strengthen causal inference, a doubly robust targeted maximum likelihood estimation (TMLE) approach [19] was also implemented, combined with a SuperLearner algorithm. The Super Learner ensemble included generalized linear models (SL.glm), penalized regression (SL.glmnet), random forests (SL.randomForest), gradient boosting (SL.xgboost), and a baseline mean-only model (SL.mean), with 10-fold cross-validation used to optimize model performance. In the presence of significant treatment-covariate interactions, stratified analyses were conducted to assess heterogeneity treatment effect (conditional average treatment effect, CATE) within relevant subgroups.

Results are expressed as adjusted/weighted odds ratio (OR) and average treatment effect (ATE) which corresponds to the estimated adjusted absolute risk difference in treatment failure percentage between exposed and unexposed patients.

Only 3 patients experienced 2 distinct episodes. Given the very small number of clustered observations, within-patient correlation was considered negligible, and models explicitly accounting for clustering (eg, GEE, mixed-effects) were not applied.

All analyses were performed in R (version 4.3.0), with a significance level set at *α* = .05. Figures were generated using GraphPad Prism (version 10.5, GraphPad Software, San Diego, CA).

## RESULTS

### Patient Characteristics

A total of 386 patients with PJI involving *Staphylococcus* spp., and/or *Corynebacterium* spp., and/or *Cutibacterium* spp. were analyzed (Figure 1). The median patient age was 72 years (IQR 65–80) and the median Charlson comorbidity index was 4 (IQR 3–6). Infections involved hip (n = 241, 62%), knee (n = 110, 28%), or shoulder (n = 35, 9%) arthroplasties. Based on the time of symptoms onset, 229 (61%) were classified as early, 33 (9%) as delayed, and 112 (30%) as late infections. Most cases of PJI (59%) were considered chronic. Fever was documented at diagnosis in 152 patients (40%; Table 1).

**Table 1.** Comparison of Characteristics According to Doxycycline Exposure

	Missing Data^a^	Total	Doxycycline		*P*-Value
			No	Yes	
		N = 386	N = 314	N = 72	
Demographic data					
Age (years), median (IQR)	…	72 (65–80)	72 (65–80)	74 (66–80)	.592
Female gender, n (%)	…	158 (41)	130 (41)	28 (39)	.696
Body mass index, median (IQR)	9/5	27 (24–33)	27 (24–33)	27 (24–33)	.695
Comorbidity					
Moderate renal insufficiency (CKD stage ≥ 3), n (%)	…	28 (7)	20 (6)	8 (11)	.162
Diabetes, n (%)	…	86 (22)	70 (22)	16 (22)	.990
Immunosuppression, n (%)	…	39 (10)	30 (10)	9 (13)	.454
Charlson comorbidity score, median (IQR)	…	4 (3–6)	4 (3–6)	4 (3–6)	.588
History of prosthetic joint infection, n (%)	…	40 (10)	30 (10)	10 (14)	.276
*Clinical characteristics*					
Prosthesis location					.799
Shoulder, n (%)	…	35 (9)	27 (9)	8 (11)	
Hip, n (%)	…	241 (62)	197 (63)	44 (61)	
Knee, n (%)	…	110 (28)	90 (29)	20 (28)	
Delay from implantation to infection	7/5				.385
Early infection (≤3 months), n (%)	…	229 (61)	183 (60)	46 (69)	
Delayed infection (3–12 months), n (%)	…	33 (9)	28 (9)	5 (8)	
Late infection (>12 months), n (%)	…	112 (30)	96 (31)	16 (24)	
Delay from inoculation to management					
Chronic infection (>4 weeks), n (%)	…	229 (59)	185 (59)	44 (61)	.732
Clinical signs					
Fever, n (%)	2/1	152 (40)	132 (42)	20 (28)	.028
Local inflammation, n (%)	3/1	259 (68)	213 (68)	46 (65)	.547
Purulent wound drainage, n (%)	4/1	231 (61)	183 (59)	48 (68)	.182
Sinus tract, n (%)	4/1	76 (20)	62 (20)	14 (20)	.957
Biological characteristics					
Neutrophils count (cells/mm^3^), median (IQR)	48/12	7 (5–10)	8 (5–11)	7 (5–9)	.109
Creatinine level (µmol/L), median (IQR)	33/9	71 (60–89)	71 (58–86)	73 (64–96)	.120
C-reactive protein (mg/L), median (IQR)	41/9	114 (51–215)	121 (55–213)	103 (35–217)	.299
Microbiological characteristics					
Blood cultures performed, n (%)	…	167 (43)	134 (43)	33 (46)	.626
Positive blood cultures, n (%)	180/39	82 (49)	69 (51)	13 (39)	.213
Polymicrobial infection, n (%)	…	161 (42)	124 (39)	37 (51)	.065
*Staphylococcus aureus,* n (%)	*…*	247 (64)	206 (66)	41 (57)	.167
Methicillin-resistant *Staphylococcus aureus,* n (%)	…	19 (5)	14 (4)	5 (7)	.369
Coagulase-negative *Staphylococcus*, n (%)	…	128 (33)	100 (32)	28 (39)	.252
*Cutibacterium* sp., n (%)	…	54 (14)	44 (14)	10 (14)	.978
*Corynebacterium* sp., n (%)	…	44 (11)	28 (9)	16 (22)	.001
Other gram-positive bacteria, n (%)	…	62 (16)	51 (16)	11 (15)	.841
Gram-negative bacilli, n (%)	…	44 (11)	38 (12)	6 (8)	.364
Anaerobic bacteria, n (%)	…	7 (2)	6 (2)	1 (1)	.999
*Management*					
Surgical management					.050
DAIR, n (%)	…	237 (61)	197 (63)	40 (56)	
One-stage exchange, n (%)	…	73 (19)	51 (16)	22 (31)	
Two-stage exchange, n (%)	…	55 (14)	47 (15)	8 (11)	
Other, n (%)	…	21 (5)	19 (6)	2 (3)	
DAIR, n (%)	…	237 (61)	197 (63)	40 (56)	.259
Optimal surgery, n (%)	…	285 (74)	226 (72)	59 (82)	.083
Empiric antibiotic therapy					
Daptomycin + piperacillin-tazobactam, n (%)	…	154 (40)	110 (35)	44 (61)	<.001
Vancomycin + third-generation cephalosporin, n (%)	…	145 (38)	129 (41)	16 (22)	.003
Other regimen, n (%)	…	87 (23)	75 (24)	12 (17)	.186
Switch antibiotic regimen					
Rifampin-based regimen^b^, n (%)	…	302 (78)	259 (82)	43 (60)	<.001
Quinolone-based regimen^b^, n (%)	…	259 (67)	233 (74)	26 (36)	<.001
>1 switch antibiotic regimen, n (%)	…	80 (21)	46 (15)	34 (47)	<.001
Management (2018–2023)	…	236 (61)	181 (58)	55 (76)	.003

Abbreviations: CKD, chronic kidney disease; DAIR, debridement, antibiotic therapy, and implant retention; IQR, interquartile range.

^a^Missing data: the notation X/Y indicates the number of missing values in the 2 respective groups (no doxycycline/doxycycline).

^b^These variables indicate whether a patient received rifampin or a quinolone as step-down therapy, either in combination with doxycycline or without doxycycline among patients in the doxycycline-exposed group. Other antibiotic regimens prescribed with or without doxycycline are detailed in Supplementary Table 1.

Perioperative cultures identified *S. aureus* in 247 patients (64%), including 19 methicillin-resistant strains. Coagulase-negative staphylococci were isolated in 128 patients (33%), *Cutibacterium* spp. in 54 (14%), and *Corynebacterium* spp. in 44 (11%). A polymicrobial etiology was observed in 161 patients (42%; Table 1).

Surgical management consisted of DAIR in 237 patients (61%), 1-stage exchange in 73 (19%), and 2-stage exchange in 55 (14%). Surgical strategy was considered optimal in 285 patients (74%).

Doxycycline was administered to 72 patients (19%; Table 1), in 45 cases (63%) as a first-line agent following empirical therapy, most often at a daily dose of 200 mg (85%, n = 41). Doxycycline was always used in combination therapy, mainly rifampin in 28 cases (39%), quinolone in 14 cases (19%), glycopeptide in 9 cases (13%; Supplementary Table 1). The median doxycycline treatment duration was 64 days (IQR 42–84), and 62 (86%) patients were treated for >30 days (Supplementary Figure 1). Rifampin was also used in 302 patients (78%) and fluroquinolones in 259 (67%).

At 12-month follow-up, treatment failure was observed in 126 cases (31% in the doxycycline exposed and 36% in the nonexposed groups), and all-cause mortality occurred in 47 cases (12%; Table 2). Approximately half of these treatment failures occurred before day 90, which corresponds to the recommended duration of antibiotic therapy following surgery for PJI (Supplementary Figure 1).

**Table 2.** Follow-Up and Treatment Outcomes of Patients According to Doxycycline Exposure

	Missing Data^a^	Total	Doxycycline		*P*-Value
			No	Yes	
		N = 386	N = 314	N = 72	
12-Month failure, n (%)	24/5	126 (35)	105 (36)	21 (31)	.453
12-Month clinical failure, n (%)	23/5	92 (26)	79 (27)	13 (19)	.191
12-Month surgical revision, n (%)	22/5	84 (23)	70 (24)	14 (21)	.592
Isolation of microorganisms from intraoperative samples, n (%)	24/5	67 (19)	55 (19)	12 (18)	.842
12-Month mortality, n (%)	0	47 (12)	41 (13)	6 (8)	.269

^a^Missing data: the notation X/Y indicates the number of missing values in the 2 respective groups (no doxycycline/doxycycline).

Only one patient exposed to doxycycline underwent surgical revision with re-identification of *Staphylococcus epidermidis*, showing doxycycline resistance that had not been documented initially.

### Characteristics and Outcomes According to Exposure to Doxycycline

Baseline characteristics, including age and Charlson comorbidity index, joint involved, infection timing, and infection type (acute vs chronic), were similar between patients exposed or not to doxycycline (Table 1). However, patients exposed to doxycycline were significantly less likely to present with fever at admission or with *Corynebacterium* spp. infections, whereas polymicrobial infections were more frequent in the doxycycline group, although this difference did not reach statistical significance (*P* = .065). Surgical strategies significantly differed between groups: 1-stage exchange was performed twice as often in the doxycycline group (31% vs 16%). Patients receiving doxycycline were also less likely to be treated with rifampin or quinolones but more likely with cotrimoxazole and glycopeptides (Table 1, Supplementary Table 1). Finally, doxycycline-exposed patients were more frequently managed during the more recent period (2018-2023) compared with nonexposed patients (Table 1).

Twelve-month treatment failure occurred in 31% of doxycycline-exposed patients and 36% of nonexposed patients, with no statistically significant difference between groups (*P* = .678; Table 2).

### Variables Associated With Treatment Failure in Univariate Analysis

Patients who experienced 12-month treatment failure were older, more frequently diabetic, and had a higher Charlson comorbidity index. They were more likely to present with fever and a sinus tract and had significantly higher C-reactive protein levels compared with those with successful outcomes (Table 3).

**Table 3.** Comparison of Patient Characteristics According to 12-Month Treatment Failure

	Missing Data^a^	Total	12-Month Success	12-Month Failure	*P*-Value
		N = 357	N = 231	N = 126	
Demographic data					
Age (years), median (IQR)	…	72 (65–80)	71 (64–78)	78 (68–83)	<.001
Female gender, n (%)	…	149 (42)	98 (42)	51 (40)	.721
Body mass index, median (IQR)	6/7	27 (24–33)	28 (24–33)	27 (23–32)	.163
Comorbidity					
Moderate renal insufficiency (CKD stage ≥ 3), n (%)	…	26 (7)	14 (6)	12 (10)	.229
Diabetes, n (%)	…	79 (22)	38 (16)	41 (33)	<.001
Immunosuppression, n (%)	…	37 (10)	20 (9)	17 (13)	.152
Charlson comorbidity score, median (IQR)	…	4 (3–6)	4 (3–5)	5 (3–7)	<.001
History of prosthetic joint infection, n (%)	…	39 (11)	20 (9)	19 (15)	.063
*Clinical characteristics*					
Prosthesis location					.077
Shoulder, n (%)	…	29 (8)	23 (10)	6 (5)	
Hip, n (%)	…	224 (63)	148 (64)	76 (60)	
Knee, n (%)	…	104 (29)	60 (26)	44 (35)	
Delay from implantation to infection	7/4				.919
Early infection (≤3 months), n (%)	…	209 (60)	137 (61)	72 (59)	
Delayed infection (3–12 months), n (%)	…	31 (9)	20 (9)	11 (9)	
Late infection (>12 months), n (%)	…	106 (31)	67 (30)	39 (32)	
Delay from inoculation to management					
Chronic infection (>4 weeks), n (%)	…	210 (59)	142 (61)	68 (54)	.169
Clinical signs					
Fever, n (%)	1/0	138 (39)	80 (35)	58 (46)	.037
Local inflammation, n (%)	2/0	239 (67)	153 (67)	86 (68)	.782
Purulent wound drainage, n (%)	3/0	213 (60)	137 (60)	76 (60)	.966
Sinus tract, n (%)	3/0	69 (19)	37 (16)	32 (25)	.037
Biological characteristics					
Neutrophils count (cells/mm^3^), median (IQR)	38/17	7 (5–10)	7 (5–10)	8 (5–11)	.236
Creatinine level (µmol/L), median (IQR)	32/9	71 (60–89)	70 (60–85)	74 (61–92)	.190
C-reactive protein (mg/L), median (IQR)	37/10	113 (52–213)	101 (35–177)	152 (72–246)	<.001
Microbiological characteristics					
Blood cultures performed, n (%)	…	156 (44)	95 (41)	61 (48)	.185
Positive blood cultures, n (%)	136/65	75 (48)	37 (39)	38 (62)	.004
Polymicrobial infection, n (%)	…	147 (41)	99 (43)	48 (38)	.382
*Staphylococcus aureus,* n (%)	*…*	226 (63)	135 (58)	91 (72)	.010
Methicillin-resistant *Staphylococcus aureus,* n (%)	…	19 (5)	7 (3)	12 (10)	.009
Coagulase-negative *Staphylococcus*, n (%)	…	119 (33)	86 (37)	33 (26)	.034
*Cutibacterium* sp., n (%)	…	49 (14)	37 (16)	12 (10)	.088
*Corynebacterium* sp., n (%)	…	42 (12)	30 (13)	12 (10)	.332
Other gram-positive bacteria, n (%)	…	55 (15)	33 (14)	22 (17)	.427
Gram-negative bacilli, n (%)	…	41 (11)	25 (11)	16 (13)	.595
Anaerobic bacteria, n (%)	…	7 (2)	2 (1)	5 (4)	.102
*Management*					
Surgical management					<.001
DAIR, n (%)	…	218 (61)	132 (57)	86 (68)	
One-stage exchange, n (%)	…	68 (19)	60 (26)	8 (6)	
Two-stage exchange, n (%)	…	53 (15)	31 (13)	22 (17)	
Other, n (%)	…	18 (5)	8 (4)	10 (8)	
DAIR, n (%)	**…**	218 (61)	132 (57)	86 (68)	.04
Optimal surgery, n (%)	**…**	266 (75)	178 (77)	88 (70)	.135
Empiric antibiotic therapy					
Daptomycin + piperacillin-tazobactam, n (%)	…	141 (39)	102 (44)	39 (31)	.015
Vancomycin + third-generation cephalosporin, n (%)	…	137 (38)	84 (36)	53 (42)	.290
Other regimen, n (%)	…	79 (22)	45 (19)	34 (27)	.103
Switch antibiotic regimen					
Rifampin-based regimen^b^, n (%)	…	280 (78)	184 (80)	96 (76)	.447
Quinolone-based regimen^b^, n (%)	…	241 (68)	159 (69)	82 (65)	.469
Doxycycline-based regimen, n (%)	…	67 (19)	46 (20)	21 (17)	.453
>1 switch antibiotic regimen, n (%)	…	77 (22)	48 (21)	29 (23)	.623
Management (2018–2023)	…	216 (61)	150 (65)	66 (52)	.020

Abbreviations: CKD, chronic kidney disease; DAIR, debridement, antibiotic therapy, and implant retention; IQR, interquartile range.

^a^Missing data: the notation X/Y indicates the number of missing values in the 2 respective groups (no 12-month failure/12-month failure).

^b^These variables indicate whether a patient received rifampin or a quinolone as step-down therapy, either in combination with doxycycline or without doxycycline among patients in the doxycycline-exposed group. Other antibiotic regimens prescribed with or without doxycycline are detailed in Supplementary Table 1.

Microbiologically, failure was associated with a higher *S. aureus* infection and bacteremia frequency and a lower coagulase-negative staphylococci frequency.

Regarding surgical management, DAIR was more common, and 1-stage exchange was less frequently performed in patients with failure. Patients with failure were also more often managed during the 2013–2017 period.

### Estimated Effect of Doxycycline on 12-Month Treatment Failure Using IPTW/SBW and TMLE Models

Overall, 307 patients (58 exposed and 249 unexposed) were included in the analysis. Of the 386 patients initially identified, 79 were excluded due to missing data on 12-month treatment failure or key covariates, or because they were considered outliers (Supplementary Figures 1, 2; Supplementary Table 2).

In the overall cohort, using the IPTW approach, doxycycline exposure was not significantly associated with the 12-month treatment failure risk (IPTW: OR = 0.72; 95% CI = .35–1.46; *P* = .354), with an adjusted absolute risk difference in treatment failure percentage (ATE) of −9% between exposed and unexposed patients (95% CI = −24% to 5%; *P* = .199).

**Figure 2.**
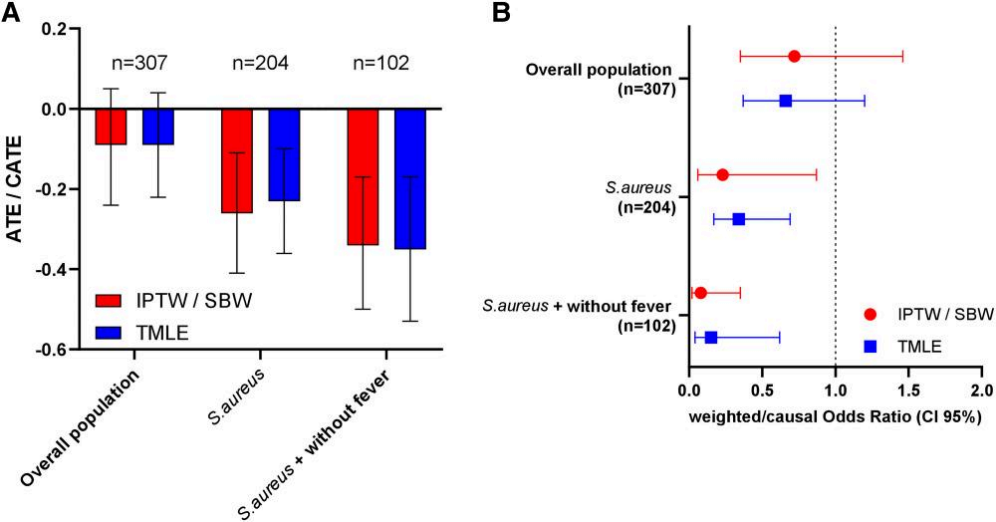
Association and effect estimates from IPTW and TMLE models assessing the impact of doxycycline on 12-month treatment failure in PJI caused by *Staphylococcus*, *Corynebacterium*, or *Cutibacterium*. (A) Interleaved bar plot of ATE estimates in the overall population and CATE estimates in clinically relevant subgroups. Bars represent causal effect estimates on the risk of failure. (B) Forest plot of odds ratio estimates for 12-month failure according to doxycycline exposure across study populations. In practice, ATE/CATE values below 0 or ORs below 1 suggest an association in favor of doxycycline, but these results are only meaningful when the 95% CI excludes 0 for the ATE or 1 for the OR. Abbreviations: ATE, average treatment effect; CATE, conditional average treatment effect; IPTW, inverse probability of treatment weighting; OR, odds ratio; PJI, prosthetic joint infection, TMLE, targeted maximum likelihood estimation.

The ATE obtained using TMLE was similar (ATE = −9%; 95% CI = −22% to 4%; *P* = .155; Figure 2; Supplementary Table 2).

Significant interactions were observed between doxycycline exposure and both *S. aureus* PJI and afebrile status at diagnosis, suggesting potential effect modification. Stratified analyses were therefore conducted to explore heterogeneity in treatment effect across these clinically relevant subgroups. These analyses revealed a significant protective association of doxycycline in 2 clinically relevant subgroups: patients infected with *S. aureus* and those without fever at diagnosis.

Among patients with *S. aureus* infection, doxycycline exposure was associated with a significant reduction in treatment failure, with an ATE of −26% (95% CI = −41% to −11%; *P* < .001) using SBW and −23% (95% CI = −36% to −10%; *P* < .001) using TMLE (Supplementary Table 2, Figure 2).

In patients without fever at diagnosis, doxycycline exposure was likewise associated with a significant absolute reduction in the risk of treatment failure (ATE = −25%; 95% CI = −39% to −11%; *P* < .001 using IPTW; ATE = −28%; 95% CI = −40% to −15%; *P* < .001 using TMLE; Supplementary Table 2, Figure 2).

Finally, among patients both infected with *S. aureus* and without fever at diagnosis, the absolute risk reduction appeared even more pronounced, ranging from 34% to 35% (ATE = −0.34; 95% CI = −.50 to −.17], *P* < .001 using SBW; ATE = −0.35; 95% CI = −.53 to −.17, *P* < .001 using TMLE; Supplementary Table 2, Figure 2).

### Sensitivity Analyses

To assess the robustness of our findings and to compare doxycycline-based regimens with the current gold standard, we performed a sensitivity analysis restricted to patients receiving rifampin–quinolone-based therapy versus those receiving doxycycline who never received the rifampin + quinolone combination at any point during their treatment. Twelve-month treatment failure occurred in 35% of doxycycline-exposed patients and 34% of nonexposed patients, with no statistically significant difference between groups (*P* = .903; Supplementary Table 3).

To further explore the role of rifampin among doxycycline-treated patients, we performed an additional sensitivity analysis restricted to patients exposed to doxycycline, comparing those receiving a rifampin backbone with those without rifampin. Twelve-month treatment failure occurred in 26% of patients receiving doxycycline–rifampin and 35% of patients receiving doxycycline without rifampin, with no statistically significant difference between groups (*P* = .432; Supplementary Table 4).

Finally, to address the potential for immortal time bias related to the timing of transition to doxycycline, we performed a sensitivity analysis excluding early treatment failures occurring within the first 3 months of therapy. Twelve-month late (3–12 months) treatment failure occurred in 25% of doxycycline-exposed patients and 22% of nonexposed patients, with no statistically significant difference between groups (*P* = .618; Supplementary Table 5).

### Doxycycline-Related Adverse Events

Adverse events occurred in only 4 of the 72 patients (6%) treated with doxycycline, including 1 case of oral mycosis, 1 case of abdominal pain, 1 case of phototoxicity, and 1 case of epilepsy.

## DISCUSSION

Our study on gram-positive PJI highlights 3 main findings. First, although not recommended as a first-line therapy, doxycycline was prescribed as part of curative antibiotic therapy in about 1 in 5 patients. Second, doxycycline exposure was not associated with the 12-month treatment failure risk and demonstrated a favorable safety profile. Third, stratified analyses showed a significant reduction in the 12-month treatment failure risk in specific subgroups, particularly patients infected with *S. aureus* and those without fever at diagnosis.

Our cohort had similar demographics to previous PJI studies, with a median age of 72 years, a predominantly male population, and two-thirds chronic infections [1, 21]. However, the higher proportion of early infections (61%) likely reflects our focus on gram-positive skin commensals, which are commonly involved in perioperative infections [7, 22].

In our cohort, doxycycline was prescribed in 19% of patients, most commonly due to bacterial resistance to other antibiotics (∼one-third), or to intolerance or allergy to first- or second-line alternatives (∼one-third). In a large French multicenter cohort, Lemaignen et al reported doxycycline use in only 3.1% of patients, mainly as suppressive therapy, with varying prescription rates depending on the pathogen (from 2.9% for methicillin-sensitive *S. aureus* to 14.5% for resistant coagulase-negative staphylococci) [2].

Our study is the largest assessing doxycycline as part of curative treatment for PJI, contributing to the limited literature beyond previous reports focused on suppressive therapy or small case series [5, 11, 23]. Rifampin remains the cornerstone of PJI treatment, primarily due to its high tissue penetration and potent activity against biofilm-associated bacteria, and quinolones are often considered the most suitable companion agents [5, 24]. However, a recent meta-analysis indicates that rifampin may only prevent a small fraction of all treatment failures [25]. Doxycycline is considered as an alternative agent in cases of allergy, intolerance, or resistance to first-line therapies, although the level of evidence supporting its efficacy remains limited [5]. It is noteworthy that a recent narrative review on the management of PJI caused by multidrug-resistant gram-positive bacteria did not mention doxycycline as a therapeutic option [24].

Our main result is that doxycycline was not associated with an increase/decrease in the 12-month treatment failure, whereas patients treated with doxycycline did not differ significantly from others in most characteristics, including involved joints, and pathogens, apart from more frequent polymicrobial and *Corynebacterium* spp. infections. For these latter, doxycycline was likely a preferred oral option due to penicillin resistance and limited alternatives like glycopeptides or daptomycin [12, 23, 24]. This overall comparability between groups minimizes potential confounding and reinforces the validity of the observed lack of association between doxycycline and 1-year outcomes. Importantly, this result was consistent across several robust statistical approaches, including IPTW/SBW, TMLE, all confirming the absence of a significant effect.

In addition, the risk of treatment failure related to acquired doxycycline resistance was low in our cohort, since only one patient exposed to doxycycline underwent surgical revision with re-identification of *S. epidermidis* with newly documented doxycycline resistance.

However, subgroup analyses suggested a reduced 12-month risk of treatment failure among patients with *S. aureus* infection and among those without fever at diagnosis. These findings suggest that doxycycline is an effective treatment option for gram-positive PJI and may be particularly suitable for *S. aureus* infections, especially when the initial presentation is afebrile.

These results align with the pharmacological advantages of doxycycline, including excellent oral bioavailability, strong bone penetration, and robust activity against biofilm-forming staphylococci [8, 9, 12]. The absence of fever in successful cases may indicate infections with a lower systemic inflammatory response or a less aggressive clinical course, scenarios in which doxycycline's unique properties, such as its ability to inhibit bacterial protein synthesis efficiently within biofilms and penetrate intracellular compartments, may create more favorable conditions for its efficacy. Indeed, the activity of ten antibiotics against *S. aureus* strains associated with PJI was assessed in both planktonic and 48-hour-old biofilm forms. While all antibiotics, including cefazolin, clindamycin, vancomycin, linezolid, nafcillin, gentamicin, and trimethoprim/sulfamethoxazole, were less effective against biofilms, only rifampin, doxycycline, and daptomycin retained significant anti-biofilm activity. Rifampin was the most effective, eradicating 90% of biofilms, followed by doxycycline (50%) and daptomycin (15%) [26]. In addition, sub-minimal inhibitory concentrations (MICs) of doxycycline have been reported to enhance *S. aureus* opsonophagocytosis, whereas gentamicin, quinolones (ofloxacin and ciprofloxacin), and vancomycin exhibited no significant effect on opsonophagocytosis by polymorphonuclear leukocytes [27–29]. In addition, a sensitivity analysis comparing patients receiving doxycycline without rifampin–quinolone to those receiving the rifampin–quinolone-based gold standard regimen showed no significant difference in 12-month treatment failure, and exclusion of early failures within the first 3 months similarly demonstrated comparable late (3–12 months) failure rates. Together, these findings support the robustness of our results and suggest that doxycycline may achieve outcomes similar to the established reference regimen.

Our study confirms doxycycline's favorable safety profile, with adverse events observed in only 4 out of 72 patients (6%), consistent with prior data from suppressive therapy, while adverse events across all antibiotic treatments are reported in 5%–50% of cases [30, 31]. In addition, among the 4 patients not included due to doxycycline exposure of <5 days (Figure 1), early discontinuation was not related to severe adverse events. The reasons were as follows: a switch based on microbiological considerations (n = 1); gastrointestinal intolerance during levofloxacin–doxycycline therapy, which resolved after substitution with cotrimoxazole (n = 1); pancytopenia attributed to vancomycin, with doxycycline administered for only one day (n = 1); and gastrointestinal intolerance during rifampin–levofloxacin therapy, persisting despite substitution with rifampin–doxycycline treatment and ultimately prompting a switch to cotrimoxazole (n = 1). Overall, given its favorable safety profile, doxycycline remains a suitable option for prolonged antibiotic therapy in bone and joint infections [12].

Finally, no treatment failures in the doxycycline group were associated with the emergence of doxycycline resistance.

Minocycline could represent an alternative in combination with rifampin because of its lower MICs against gram-positive pathogens and fewer pharmacokinetic interactions [32–34]. However, the favorable outcomes observed in this study were achieved with doxycycline, and limited data in the literature support the use of minocycline in this setting.

Despite these promising findings, several limitations must be acknowledged. First, as an observational study, our results are susceptible to residual confounding, even though we applied robust causal inference methods (IPTW/SBW and TMLE), and adjusted for relevant covariates based on a DAG. Second, although stratified analyses revealed significant benefits, the relatively small sample sizes in these subgroups limit statistical power and generalizability, as reflected by the wide confidence intervals in some estimates. Third, doxycycline was more frequently prescribed in recent years and in combination with specific surgical strategies, such as 1-stage exchange, which may themselves be associated with better outcomes. Although we adjusted for calendar period and surgical approach, unmeasured changes in practice over time may still have influenced our results. To our knowledge, our study is the largest assessing doxycycline as part of curative treatment for PJI, contributing to the limited literature beyond previous reports focused on suppressive therapy or small case series [5, 11, 23].

In conclusion, the use of doxycycline in combination with other antibiotics was not associated with treatment failure at 12-month in the overall cohort of PJI caused by *Staphylococcus*, *Corynebacterium*, or *Cutibacterium* and with a favorable tolerability profile. Moreover, doxycycline may represent a valuable treatment option for the management of *S. aureus* PJI, particularly in cases of intolerance, resistance, or drug interactions with rifampin or quinolones. It is also especially relevant for corynebacterial PJI, where therapeutic options are often very limited, particularly in polymicrobial infections, which are common in this context. Unlike previous studies, our work provides a comparative perspective and focuses on curative treatment, with careful adjustment for confounding factors. These findings may help guide clinical decision-making when guideline-recommended therapies are not feasible and could support a stronger recommendation for doxycycline as an alternative in the curative treatment of PJI.

## Supplementary Material

ofag098_Supplementary_Data

## Supplementary Data

Supplementary materials are available at *Open Forum Infectious Diseases* online. Consisting of data provided by the authors to benefit the reader, the posted materials are not copyedited and are the sole responsibility of the authors, so questions or comments should be addressed to the corresponding author.
