# Supplementary material for: Effectiveness of Doxycycline in Combination With Other Antibiotics for Gram-Positive Periprosthetic Joint Infections: A Causal Inference Study
Source: Open Forum Infect Dis. 2026 Feb 25;13(3):ofag098. doi: 10.1093/ofid/ofag098 (PMC12968137; doi:10.1093/ofid/ofag098)
Supplement: ofag098_Supplementary_Data [file ofag098_supplementary_data.docx]

**Effectiveness of Doxycycline in Combination with Other Antibiotics for Gram-Positive Periprosthetic Joint Infections: a Causal Inference Study.**

**Supplemental Figure 1.** Bar Graph showing patient distribution across doxycycline prescription durations

**Supplemental Figure 2. Kaplan-Meier Curve of One-Year Treatment Failure in Patients with Prosthetic Joint Infection who experienced one-year treatment failure.**

The vertical line at day 90 represents the end of the standard antibiotic treatment period for prosthetic joint infections following surgical management.


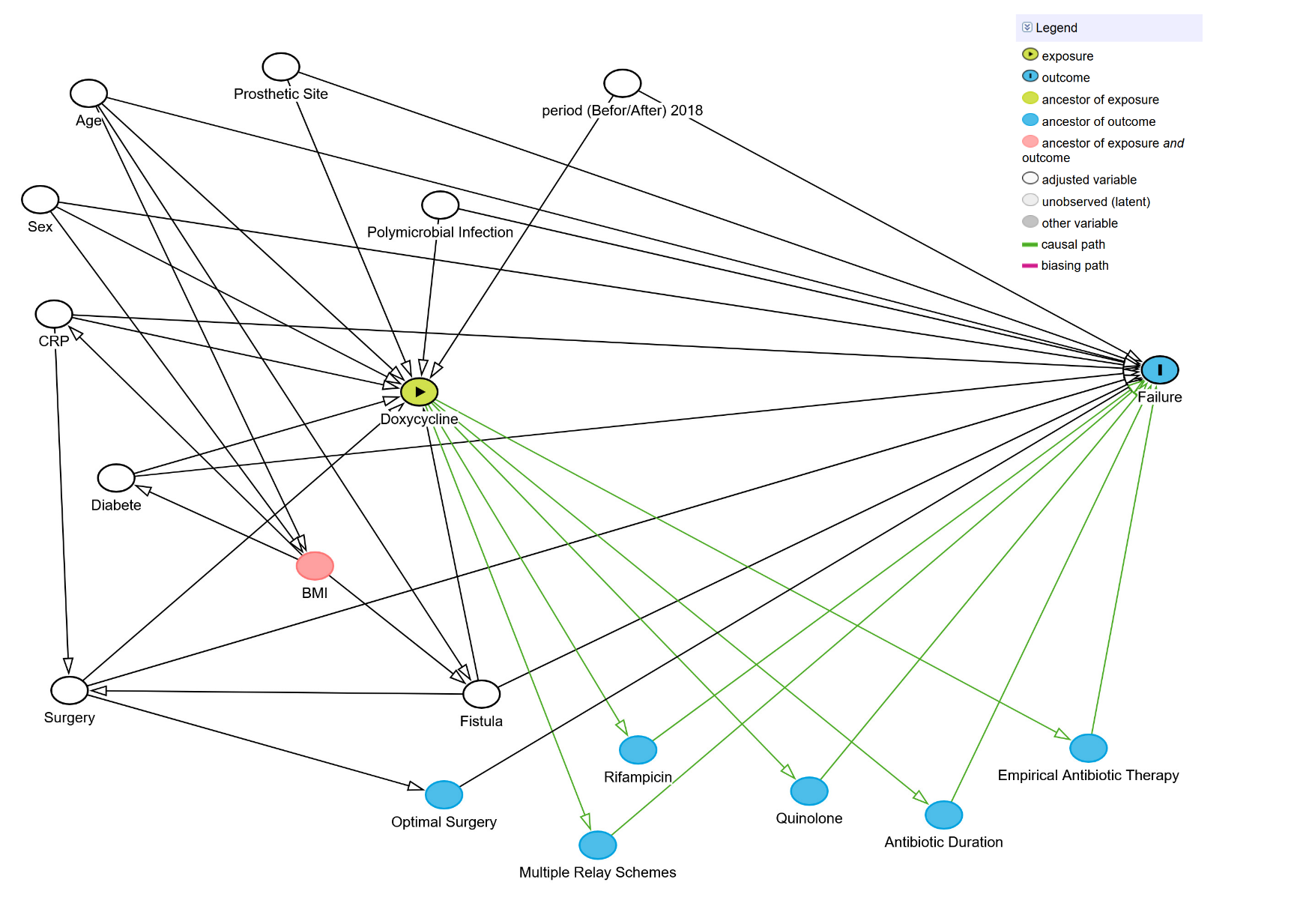


**Supplemental Figure 3.** Selection of adjustment variables using Directed Acyclic Graphs (DAGs)


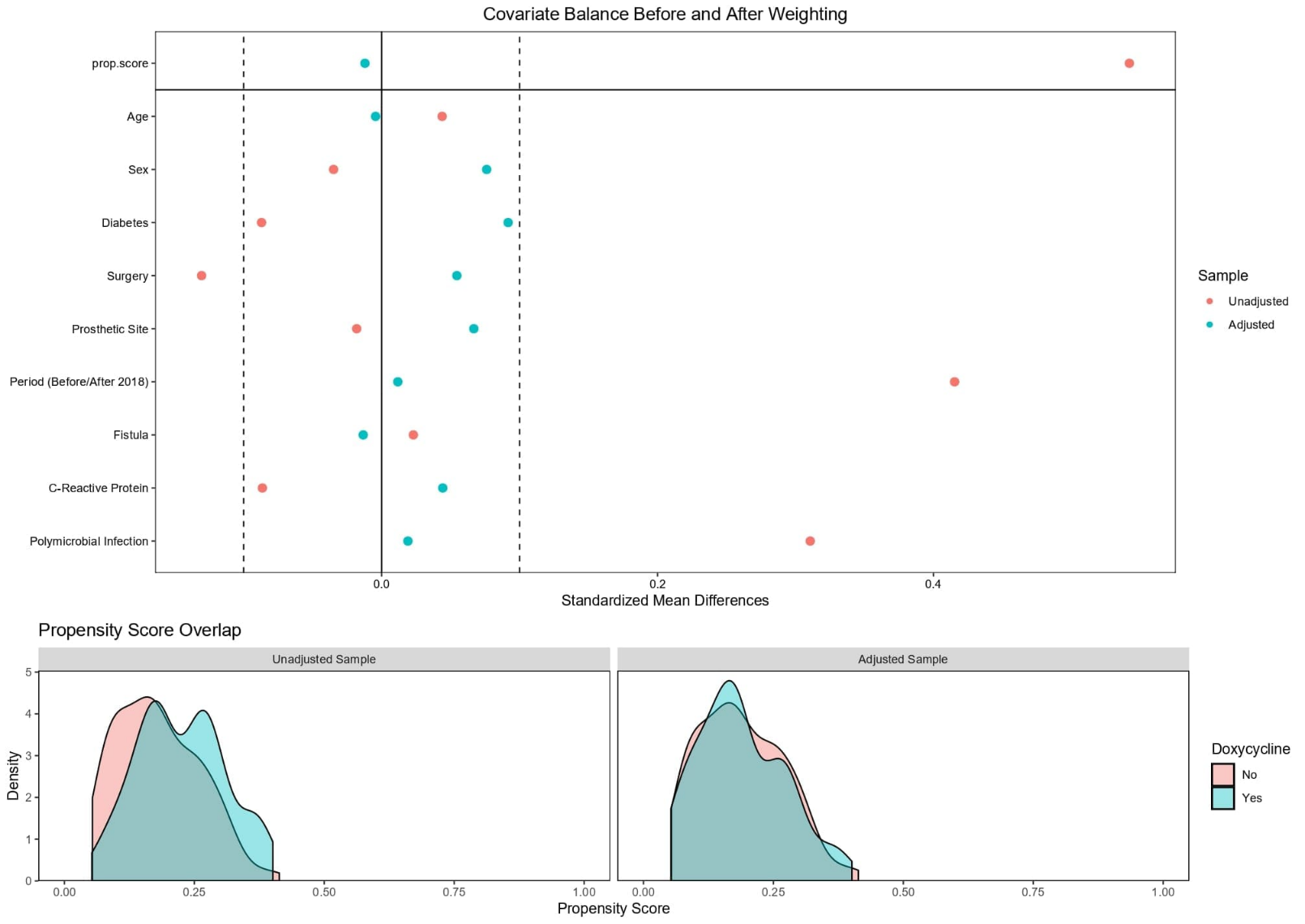


**Supplemental Figure 4.** Covariate Balance Assessment After Propensity Score Weighting in the Overall Population

**Supplemental Table 1. Antibiotics used as step-down therapy based on culture results**

|  | **Antibiotics used as step-down therapy based on culture results** | | | | **Adjunctive antibiotic to doxycycline** |
| --- | --- | --- | --- | --- | --- |
|  | **Total** | **Doxycycline** | | **P-value** | **Doxycycline** |
|  |  | **no** | **yes** |  | **yes** |
|  | **N=386** | **N=314** | **N=72** |  | **N=72** |
| Penicillin A ± clavulanic acid | 45 (12) | 39 (12) | 6 (8) | 0.33 | 3 (4) |
| Intravenous penicillin M or first-generation cephalosporin | 21 (5) | 17 (5) | 4 (6) | 1.00 | 1 |
| Intravenous third-generation cephalosporin | 1 | 1 | 0 | 1.00 | 0 |
| Intravenous broad-spectrum β-lactamin | 8 (2) | 7 (2) | 1 (1) | 1.00 | 1 |
| Fluoroquinolone | 259 (67) | 233 (74) | 26 (36) | <0.001 | 14 (19) |
| Rifampin | 302 (78) | 259 (82) | 43 (60) | <0.001 | 28 (39) |
| Clindamycin or pristinamycin | 30 (8) | 21 (7) | 9 (13) | 0.097 | 9 (13) |
| Cotrimoxazole | 31 (8) | 21 (7) | 10 (14) | 0.043 | 8 (11) |
| Fucidic acid | 11 (3) | 6 (2) | 5 (7) | 0.055 | 3 (4) |
| Metronidazole | 8 (2) | 7 (2) | 1 (1) | 0.430 | 1 |
| Glycopeptide | 20 (5) | 10 (3) | 10 (14) | <0.001 | 9 (13) |
| Lipopeptide | 16 (4) | 12 (4) | 4 (6) | 0.735 | 1 |
| Oxazolidinone | 5 (1) | 4 (1) | 1 | 1.000 | 0 |
| Others* | 3 (1) | 3 (1) | 0 | 0.929 | 0 |
| *colistin (n=1), cefuroxime (n=2) |  |  |  |  |  |

**Supplemental Table 2.** Association and Effect Estimates from IPTW / TMLE Models across study populations

| **Population** | **Method** | **Estimator** | **Value** | **CI_inf** | **CI_sup** | **P value** |
| --- | --- | --- | --- | --- | --- | --- |
| Overall population | IPTW | **OR** | 0.72 | 0.35 | 1.46 | 0.3544 |
|  | IPTW | **ATE** | -0.09 | -0.24 | 0.05 | 0.1990 |
|  | TMLE | **OR** | 0.66 | 0.37 | 1.20 | 0.1741 |
|  | TMLE | **ATE** | -0.09 | -0.22 | 0.04 | 0.1552 |
|  |  |  |  |  |  |  |
| *Staphylococcus aureus ± without fever  n=278* | IPTW | **OR** | 0.34 | 0.15 | 0.75 | **0.0075** |
|  | IPTW | **ATE** | -0.22 | -0.35 | -0.08 | **0.0013** |
|  | TMLE | **OR** | 0.41 | 0.23 | 0.75 | **0.0038** |
|  | TMLE | **ATE** | -0.19 | -0.30 | -0.07 | **0.0011** |
|  |  |  |  |  |  |  |
| *Staphylococcus aureus  n=204* | SBW | **OR** | 0.23 | 0.06 | 0.87 | **0.0297** |
|  | SBW | **ATE** | -0.26 | -0.41 | -0.11 | **0.0008** |
|  | TMLE | **OR** | 0.34 | 0.17 | 0.69 | **0.0025** |
|  | TMLE | **ATE** | -0.23 | -0.36 | -0.10 | **0.0005** |
|  |  |  |  |  |  |  |
| Without fever  n= 176 | IPTW | **OR** | 0.24 | 0.09 | 0.69 | **0.0083** |
|  | IPTW | **ATE** | -0.25 | -0.39 | -0.11 | **0.0004** |
|  | TMLE | **OR** | 0.20 | 0.08 | 0.54 | **0.0013** |
|  | TMLE | **ATE** | -0.28 | -0.40 | -0.15 | **0.0000** |
|  |  |  |  |  |  |  |
| Staphylococcus aureus + without fever  n=102 | SBW | **OR** | 0.08 | 0.02 | 0.35 | **0.0013** |
|  | SBW | **ATE** | -0.34 | -0.50 | -0.17 | **0.0001** |
|  | TMLE | **OR** | 0.15 | 0.04 | 0.62 | **0.0090** |
|  | TMLE | **ATE** | -0.35 | -0.53 | -0.17 | **0.0001** |
|  |  |  |  |  |  |  |

*CI_inf / CI_sup: lower / upper bound of the 95% confidence interval*

***IPTW****: Inverse Probability of Treatment Weighting;* ***SBW****: Stable Balancing Weights,* ***TMLE*** *: Targeted Maximum Likelihood Estimation*

**Supplemental Table 3.** Sensitivity analysis comparing follow-up and treatment outcomes between doxycycline without rifampin–fluoroquinolone and rifampin–fluoroquinolone–based regimens (n=272 patients)

|  | **Missing data #** | **Total** | **rifampin-quinolone = yes doxycycline = no** | **doxycycline = yes rifampin-quinolone = no** | **P-value** |
| --- | --- | --- | --- | --- | --- |
|  |  |  |  |  |  |
|  |  | **N=272** | **N=220** | **N=52** |  |
| 12-month failure, n (%) | 16/4 | 87 (35) | 70 (34) | 17 (35) | 0.903 |
| Clinical recurrence of infection, n (%) | 16/4 | 65 (26) | 54 (26) | 11 (23) | 0.613 |
| Surgical revision, n (%) | 16/4 | 60 (24) | 48 (24) | 12 (25) | 0.830 |
| 12-month mortality, n (%) | 0 | 28 (10) | 23 (10) | 5 (10) | 0.858 |

# missing data: the notation X/Y indicates the number of missing values in the two respective groups.

**Supplemental Table 4.** Sensitivity analysis of follow-up and treatment outcomes restricted to doxycycline-treated patients: with versus without rifampin (n=72 patients)

|  | **missing data #** | **Total** | **rifampin** | | **P-value** |
| --- | --- | --- | --- | --- | --- |
|  |  |  | **no** | **yes** |  |
|  |  | **N=72** | **N=44** | **N=28** |  |
| 12-month failure, n (%) | 4/1 | 21 (31) | 14 (35) | 7 (26) | 0.432 |
| clinical recurrence of infection, n (%) | 4/1 | 13 (19) | 9 (23) | 4 (15) | 0.642 |
| surgical revision, n (%) | 4/1 | 14 (21) | 9 (23) | 5 (19) | 0.694 |
| 12-month mortality, n (%) | 0 | 6 (8) | 4 (9) | 2 (7) | 1.000 |

# missing data: the notation X/Y indicates the number of missing values in the two respective groups (no rifampin/rifampin).

**Supplemental Table 5.** Sensitivity analysis comparing late (3–12 months) treatment outcomes between patients exposed to doxycycline and those not exposed (n=326 patients)

|  | **missing data #** | **Total** | **doxycycline** | | **P-value** |
| --- | --- | --- | --- | --- | --- |
|  |  |  | **no** | **yes** |  |
|  |  | **N=326** | **N=260** | **N=66** |  |
| Late 12-month failure, n (%) | 24/5 | 66 (22) | 51 (22) | 15 (25) | 0.618 |
| Late clinical recurrence of infection, n (%) | 23/5 | 50 (17) | 39 (16) | 11 (18) | 0.769 |
| Late surgical revision, n (%) | 22/5 | 50 (17) | 38 (16) | 12 (20) | 0.489 |
| Late 12-month mortality, n (%) | 0 | 18 (5) | 16 (6) | 2 (3) | 0.490 |

# missing data: the notation X/Y indicates the number of missing values in the two respective groups (no doxycycline/doxycycline)
